# Pretreatment with Human Chorionic Gonadotropin Protects the Neonatal Brain against the Effects of Hypoxic-Ischemic Injury

**DOI:** 10.3389/fped.2017.00232

**Published:** 2017-11-03

**Authors:** Tammy Z. Movsas, Rebecca L. Weiner, M. Banks Greenberg, David M. Holtzman, Rafael Galindo

**Affiliations:** ^1^Zietchick Research Institute, Plymouth, MI, United States; ^2^Department of Pediatrics and Human Development, Michigan State University College of Human Medicine, East Lansing, MI, United States; ^3^Department of Neurology, Hope Center for Neurological Disorders, Washington University, St. Louis, MO, United States

**Keywords:** cerebral palsy, human chorionic gonadotropin, fetal brain, ischemia, neuroprotection, brain injury, excitotoxicity, chorionic gonadotropin

## Abstract

**Introduction:**

Though the human fetus is exposed to placentally derived human chorionic gonadotropin (hCG) throughout gestation, the role of hCG on the fetal brain is unknown. Review of the available literature appears to indicate that groups of women with higher mean levels of hCG during pregnancy tend to have offspring with lower cerebral palsy (CP) risk. Given that newborn cerebral injury often precedes the development of CP, we aimed to determine whether hCG may protect against the neurodegenerative effects of neonatal brain injury.

**Methods:**

We utilized the Rice–Vannucci model of neonatal cerebral hypoxia-ischemia (HI) in postnatal day 7 mice to examine whether intraperitoneal administration of hCG 15–18 h prior, 1 h after or immediately following HI decrease brain tissue loss 7 days after injury. We next studied whether hCG has pro-survival and trophic properties in neurons by exposing immature cortical and hippocampal neurons to hCG *in vitro* and examining neurite sprouting and neuronal survival prior and after glutamate receptor-mediated excitotoxic injury.

**Results:**

We found that intraperitoneal injection of hCG 15 h prior to HI, but not at or 1 h after HI induction, resulted in a significant decrease in hippocampal and striatal tissue loss 7 days following brain injury. Furthermore, hCG reduced *N*-methyl-d-aspartate (NMDA)-mediated neuronal excitotoxicity *in vitro* when neurons were continuously exposed to this hormone for 10 days or when given at the time and following neuronal injury. In addition, continuous *in vitro* administration of hCG for 6–9 days increased neurite sprouting and basal neuronal survival as assessed by at least a 1-fold increase in MAP2 immunoreactivity and a 2.5-fold increase in NeuN + immunoreactivity.

**Conclusion:**

Our findings suggest that hCG can decrease HI-associated immature neural degeneration. The mechanism of action for this neuroprotective effect may partly involve inhibition of NMDA-dependent excitotoxic injury. This study supports the hypothesis that hCG during pregnancy has the potential for protecting the developing brain against HI, an important CP risk factor.

## Introduction

Human chorionic gonadotropin (hCG) is a placentally derived hormone produced by the syncytiotrophoblast following implantation. The human fetus is exposed to hCG throughout its intrauterine development (1). Back in early 1980s, hCG receptors (also known as lutropin receptors) were identified in multiple fetal organs such as kidney, lung, and colon (2, 3), thus suggesting hCG’s role in fetal/neonatal growth and differentiation (4–7). However, little progress has been made over the years in defining this developmental role (4–7). Studies have shown that hCG can cross the blood–brain barrier, and that hCG receptors exist in fetal and neonatal rat brains (4–7). *In vitro* studies have also demonstrated that hCG decreases neuronal cell death, promotes the outgrowth of fetal neuronal cell processes, and supports the proliferation of neural stem cells (5, 8, 9). Furthermore, hCG can decrease ischemic brain injury in adult rodent models of stroke and it has been shown to improve functional recovery following spinal cord injury in rats (8, 10–14). Given the above evidence, hCG may act as neuroprotectant against the neurodegenerative effects of neonatal cerebral hypoxia-ischemia (HI).

Hypoxia-ischemia has been identified as a major cerebral palsy (CP) risk factor (15–18). Given that almost 90% of the CP-associated brain injuries occur *in utero* or around the time of birth (19, 20), if hCG does indeed protect against hypoxic-ischemic brain injury, we would expect the offspring of women with increased levels of hCG to have a lower risk of CP. To explore this potential relationship, we searched the medical literature for studies pertaining to maternal hCG levels during pregnancies of singleton births and identified several distinct groups of women with decreased mean hCG levels during pregnancy. We then searched the literature for studies of CP risk for offspring born to each of these distinct groups. We found that that the groups of women with lower mean serum hCG tended to be at higher risk for having children with CP. For example, mean maternal hCG levels are significantly lower in women bearing boys, American women of Asian-descent, women bearing boys with cryptorchidism and women who are obese (compared with women bearing girls, American women of non-Asian descent, women bearing boys without cryptorchidism, and women of normal weight, respectively) (21–25). These same groups of women have increased CP risk for their offspring compared with their counterparts (26–34). Thus, we indirectly identified a potential inverse hCG–CP relationship which provides support to the hypothesis that hCG has neuroprotective actions in the immature brain and may play a role in diminishing CP risk.

In the present work, we sought to directly examine whether hCG can serve as a neuroprotectant against the CP risk factor of neonatal HI. We first utilized the Rice–Vannucci model of mouse neonatal HI and investigated whether intraperitoneal (IP) hCG administration to postnatal day 7 (P7) mouse pups at three treatment time points (15 h prior to hypoxia, 1 h prior to hypoxia and immediately after hypoxia) decreases cerebral tissue loss 7 days following injury. We then asked whether *in vitro* hCG exposure favors the survival and growth of immature neurons and protects them against *N*-methyl-d-aspartate (NMDA)-dependent excitotoxic injury. To explore this second aim, dissociated immature hippocampal and/or cortical neurons were continuously exposed to hCG for one to up to 10 days, while we examined its effects on neuronal survival and neurite sprouting prior and after glutamate-mediated excitotoxic injury. Altogether, we found that peripheral pretreatment with hCG reduces the effects of neonatal cerebral HI *in vivo* and that direct hCG exposure to neurons favors neuronal growth and survival under baseline and injurious conditions. The results described here support the hypothesis that hCG can act as a neuroprotectant in the developing brain. Future studies should aim at further exploration of the role of hCG in brain development as well as the mechanisms involved in hCG-mediated neuroprotection.

## Materials and Methods

### Animals and Surgical Procedures

C57BL/6 (Charles River Laboratories Inc., Roanoke, IL, USA) mice were kept under 12/12 light/dark cycles with *ad libitum* access to food and water. Neonatal hypoxic-ischemic brain injury was performed in male and female mouse pups at P7 as previously described (35). Only pups with a body weight greater than 3.0 g at the time of carotid surgery (P7) were used in this study. Briefly, pups were anesthetized using 3% halothane for induction and 2.5% halothane for maintenance (balance room air). Under anesthesia, blood flow through the left carotid artery was permanently interrupted by carotid artery cauterization through an incision in the neck. Following carotid surgery, pups were returned to the dam and allowed to recover for a minimum of 3 h. Pups were then placed in pairs into individual thermoregulated hypoxia cylindrical chambers for 45 min at 36.5°C with 8% oxygen. Each chamber contained a control pup and hCG-treated pup. High-grade humidified 8% oxygen (±0.02%) balanced with nitrogen was used. After completion of the hypoxia period, pups were returned to the dam until the day they were euthanized at postnatal day 14 (P14). In order to avoid potential confounders related to changes in body temperature or other environmental conditions, for each experiment, the saline control and hCG-treated animals were exposed at the same time to the hypoxia chamber and to the same temperature conditions and for the same duration of time. Similarly, every attempt was made to equalize the surgical and post-surgical recovery conditions of the control and hCG-treated groups.

### hCG and Vehicle Injections

For all *in vivo* and *in vitro* experiments described below (except as noted), the source of hCG was Sigma-Aldrich (St. Louis MO, USA, Catalog no. CG10). When noted that a different source of hCG was utilized, the source was Sigma-Aldrich (St. Louis MO, USA, Catalog no. C0434). Reconstitution of hormone powder was done in phosphate-buffered saline (PBS) as per manufacturer’s recommendations to a concentration of 10,000 IU/mL. Drug was then diluted to the specified concentrations in the corresponding control *in vitro* (control salt solution or neurobasal media) and *in vivo* (0.9% normal saline, NS) solutions.

A single 1,500 IU/kg IP injection of hCG was administered to pups exposed to 45 min of hypoxia after carotid ligation and at three different time points: 15 h prior to hypoxia, 1 h prior to hypoxia, or immediately after hypoxia. NS-injected littermates were used as vehicle controls and exposed to the injury paradigm at the same time as the neonatal pups injected with hCG. Every attempt was made to segregate an equal number of males (M) and females (F) to each treatment group to account for gender-based bias (total M/F; NS 18/14 and hCG 18/12). Drug or vehicle was injected into the IP space using a 30-G needle attached to a 100 μL Hamilton syringe (Hamilton, Reno, NV, USA). Given hCG’s long half-life of about 36 h (1, 36, 37), we chose to administer a single IP dose to minimize potential toxic additive effects of hCG. A dosage of 1,500 IU/kg of hCG was given since that is the dose that was shown to be effective and safe in adult murine stroke models (8).

### Histology and Tissue Injury Quantification

At 7 days following HI, P14 mice pups were deeply anesthetized with pentobarbital (150 mg/kg) and perfused transcardially with ice-cold PBS containing 3 U/mL heparin. Brains were removed and immersion-fixed in 4% (w/v) paraformaldehyde (PFA) in 0.1 M phosphate buffer (pH 7.4) at 4°C for 24 h, and then cryoprotected in 30% (w/v) sucrose in 0.1 M phosphate buffer until freezing in powdered dry ice and sectioned into 50 µm coronal sections with a freezing sliding microtome. The uninjured, contralateral right hemisphere was marked by nicking it with a razor blade. A set of coronal brain sections (spaced 50 µM apart) beginning at the genu of the corpus extending to the mid body of the dorsal hippocampus were mounted in DAPI mounting medium (Vector Labs) and imaged *via* Nanozoomer (Hamamatsu Photonics). Percentage of tissue loss was calculated by comparing the cortex, striatum, and hippocampus of the injured hemisphere (left) to the uninjured hemisphere (right) using the Nanozoomer imaging software as previously described (38, 39). Three coronal sections per quantification area per animal were used for each of the brain regions studied. Striatum volume was calculated using those sections containing the striatum at the level of the genu of the corpus callosum. Hippocampal injury was determined by analyzing sections containing all layers of the dorsal hippocampal formation. Cortical areas were analyzed on the sections that contained the dorsal hippocampus. The investigators were blinded to the treatment during area analysis.

### Determination of Neonatal Mouse Body Temperature

Body temperature recordings were obtained by transiently introducing a miniature thermistor recording probe coated with triple antibiotic lubricating gel (TH-10Kmp thermistor, Cell MicroControls, Norfolk, VA, USA) into the rectum of a P7 C57BL/6 mouse and connected to a 2 channel micro temperature controller (mTCII, Cell MicroControls, Norfolk, VA, USA). For the determination of body temperature during group huddle (Figure 2A), a consistent five-mouse pup huddle was used. Animals were injected intraperitoneally with 0.9% NS-control solution or 1,500 IU/kg of hCG 1 h or 15–18 h prior to temperature recording. Recording of huddle pup temperature was done immediately and approximately within 20 s after the animal was taken out of the group huddle. For the recording of body temperature at a constant physiological temperature of 36.5°C and following removal from physiological to room temperature (Figures 2B–D), P7 pups treated with hCG (1,500 IU/kg) or 0.9% NS vehicle 15–18 h prior to temperature recording were briefly anesthetized under halothane and the thermistor mini recording probe was introduced into the rectum. Subsequently, the probe was temporarily secured with adhesive tape to the proximal portion of the mouse tail. The pups were then allowed to fully recover from anesthesia for 30 min before any temperature recording. Anesthesia recovery and temperature equilibration were made in a thermoregulated hypoxia chamber set at a constant temperature of 36.5°C and supplied by a constant flow of humidified 21% FiO_2_ air. Following the 30-min post-anesthesia period, the rectal temperature was recorded every minute for 10 min followed by the animal being removed from the thermoregulated 36.5°C chamber and into chamber set at a constant room ambient temperature of 23°C in 21% FIO_2_. In this chamber, the temperature was continuously recorded every minute for 30 min before the probe was removed. In order to minimize potential inter-recording variations among the control and hCG-treated groups, each single-temperature recording session was carried in parallel using a control, saline-treated, pup paired with an hCG-treated neonate. Every attempt was made to segregate an equal amount of males and females to each group (Control: 6 females/6 males; 1 h post-hCG: 3 females/2 males; 15–18 h post-hCG: 6 females/6 males). Postnatal day 7 mouse pups only weighing 3 g or more were included in these experiments and the average weight did not significantly differ among groups (Control: 3.5 ± 0.10 g, *N* = 12; 1 h post-hCG: 3.4 ± 0.13 g, *N* = 5; 15–18 h hCG, 3.6 ± 0.14 g, *N* = 12).

### Determination of Serum hCG Concentration

Serum was extracted from P7 pups *via* cardiac puncture 1 h or 15–18 h following IP hCG administration. Approximately 50–70 µL of blood was obtained from each pup. Blood samples were immediately placed in serum separating cold microcentrifuge tubes and centrifuged at 10,000 rpm for 15 min. Serum samples were then loaded at 1:15 dilution into a 96-well commercially available sandwich hCG enzyme-linked immunosorbent assay (ELISA; ab100533, Abcam, Cambridge, MA, USA). Standard curve and serum samples were probed using the ELISA kit in duplicate and according to the manufacturer’s instructions. Conversion from ng/mL to IU/mL was determined using this assay and generating a second dilution curve from a known concentration of the hCG employed in the animal and cell experiments (CG10, Sigma, St. Louis, MO, USA).

### Dissociated Cortical and Hippocampal Cultures and Ibotenic Acid-Mediated Excitotoxic Assay

The cortex and hippocampus were microdissected from embryonic day 18 C57BL/6 embryos and incubated in Hank’s balanced salt solution with 2.5% (10×) trypsin for 7 min at 37°C. Following trypsin incubation, cells were dissociated into a homogenous suspension by gentle pipetting tissue up and down 15–20 times utilizing a sterile 1mL pipette. The dissociated neurons of cortices and hippocampi were resuspended after cold centrifugation (4°C; 1,500 rpm) in neurobasal media and placed in 24-well plates precoated with Poly-l-Lysine. To avoid plating neurons at different densities in different wells, cells were plated at the same time from a common dissociated neuronal preparation at a calculated equal neuronal concentration of 100,000 cells/well; all neurons for a given experiment were plated simultaneously and treated equally prior to any drug or injury treatment. Although we did quantify the amount of neurons in each well, routine visual inspection of neurons under phase contrast microscopy prior to exposure to any form of treatment appeared to show consistent density, growth, and morphology across all wells. Cells were maintained in neurobasal media plus 1X B-27 supplement, 0.5-mM l-glutamine with penicillin-streptomycin, and 50% of the media was replaced every 3–4 days. Neurons were examined in the presence or absence of hCG (diluted in neurobasal media at a concentration of 2 IU/mL unless otherwise specified). At DIV12, cultured neurons were washed with a controlled salt solution (CSS) containing 120-mM NaCl, 5.4-mM KCl, 1.2 mM CaCl_2_, 15-mM glucose, and 25-mM Tris–HCl, pH = 7.40, at room temperature. Magnesium was omitted to avoid blockade of the NMDA receptor channels. Following the wash, cells were exposed to 50-µM Ibotenic acid (IBO) with 100-µM glycine in CSS or CSS alone for 5 min then washed and incubated in neurobasal media for 24 h prior to lactase dehydrogenase (LDH) assay or cytological assessment as previously described (40).

### Lactate Dehydrogenase Assay

At 24-h post-excitotoxic injury with IBO or prior to PFA fixation, media from individual wells of cortical and hippocampal neuron cultures were collected and assayed for LDH using a cytotoxicity detection kit (Roche) as previously described (38). Briefly, 100 µL of culture medium per well/sample in duplicate was incubated with gentle shaking with the kit reagents for 30 min in the dark, after which the product was measured at 490 nm utilizing a standard 96-well microplate reader (Bio-Tek Instruments). Following collection of conditioned media for LDH, cells were fixed with 4% PFA in phosphate buffer for 30 min at room temperature. After washing with PBS, cells were permeabilized with PBS-X, blocked in normal serum, and probed for immunocytochemistry.

### *In Vitro* Immunofluorescence Quantification

Quantification of neurons and neuronal processes was achieved by staining PFA-fixed cortical and hippocampal neurons with a mouse MAP2 antibody (1:1,000, Millipore) or NeuN Alexa Fluor^®^488 conjugated antibody (1:100, Millipore). Sample digital images of MAP2 and NeuN stained neurons in control and hCG-treated neuronal cultures were obtained. For quantification, the analysis area (1 mm × 1 mm region) per digital image and the number of images and image characteristics (3–4 images/neuronal culture well) in the culture well were the same for each experiment and treatment condition. Every attempt was made to select the same approximate location in the culture for each experimental condition in a treatment-blinded fashion. Areas were chosen away from the edge of the 1.6-cm culture and at approximately the 3, 6, 9, and 12 o’clock location. The amount of immunofluorescence was quantified utilizing ImageJ analysis software by thresholding the immunofluorescence of control conditions and automating the analysis of NeuN or MAP2 immunofluorescence per region for all cultures while keeping the same threshold and image settings (41). Data were expressed as the average amount of immunofluorescence per fixed unit area. A single *N*-value represents average stain quantified per area per a single culture well.

### Chronic hCG Exposure/IBO Assay Procedure

Dissociated cortical and hippocampal late embryonic (E18) neurons were exposed to 2 IU/mL of hCG beginning at 3 days (DIV3) after plating and continuing until 24 h post-injury at DIV13 (totaling 10 days of hCG exposure). Excitotoxic injury was achieved by transient, 5-min, 50-µM IBO administration at DIV12 as previously described (40). An hCG concentration of 2 IU/mL was first chosen given that this is the calculated average concentration of hCG present in human fetal amniotic fluid (42–44). Neuronal cell viability was measured by quantifying the amount of neurite degeneration *via* MAP2 staining 24 h post-IBO administration and by examining lactate dehydrogenase activity (described above) in the culture media 24 h following injury.

### Acute hCG Exposure/IBO Assay Procedure

Dissociated E18 cortical neurons were allowed to grow for 12 days in the absence of hormone and then were transiently exposed to 50-µM IBO for 5 min in the absence and presence of hCG at concentration of 2 and 20 IU/mL. Control neurons were exposed only to the control salt solution. hCG exposure continued for 24 h after injury when the neurons were fixed in PFA and processed for MAP2 immunocytochemistry.

### High-Dose hCG Exposure

In a separate experiment and in order to evaluate the effects of high-dose hCG on neuronal cell count and neurite sprouting, we continuously exposed neurons for 6 days to 2, 10, and 100 IU/mL of hCG diluted in culture media starting at DIV3. After the hCG-exposure period, neurons were fixed in PFA and processed for MAP2, NeuN and GFAP immunocytochemistry.

### Statistics

Data are presented as mean ± SEM and were compared using one-way ANOVA followed by Bonferroni test for multiple comparisons, one-sample *t*-test for hypothetical value comparisons, and one or two-tailed Student’s *t*-test for two-group comparisons unless otherwise specified. Statistical significance was set at 0.05 where *p < 0.05 and **p < 0.01. Statistics were performed using GraphPad Prism (GraphPad software).

## Results

### Pretreatment with hCG Protects the Immature Brain from Hypoxic-Ischemic Neonatal Neurodegeneration

**Figure 1:**
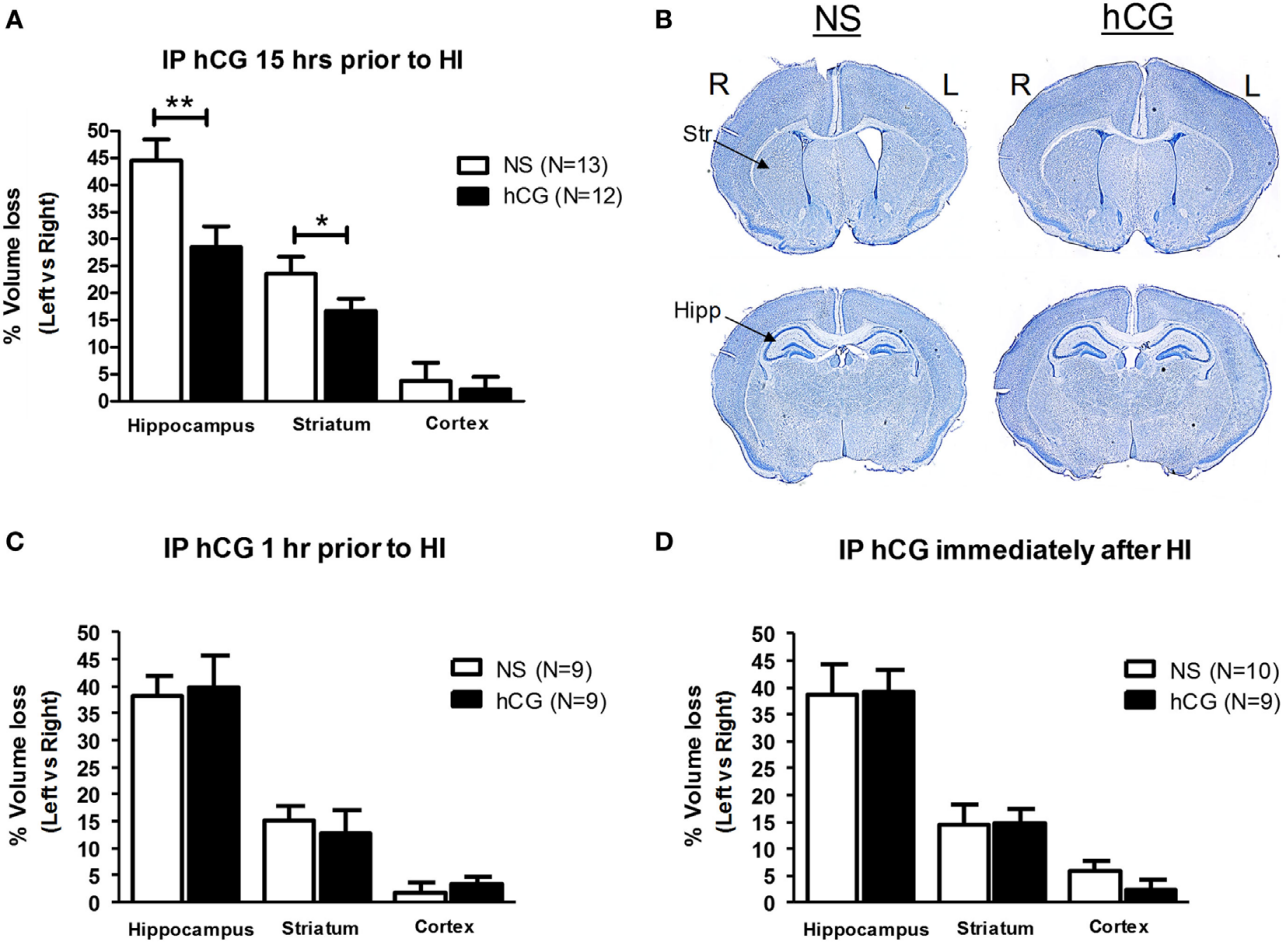
*In vivo* hCG IP administration protects the brain from the neurodegenerative effects of HI in a time-dependent manner. **(A)** The relative tissue losses [calculated by the percent difference between left (L) ipsilateral, ischemic, and right (R) contralateral, non-ischemic, hemisphere] in the hippocampus, striatum and cerebral cortex 7 days after carotid ligation followed by 45 min of hypoxia from NS-injected vs. hCG-injected (1,500 IU/kg) P7 mouse pups treated 15 h prior to HI. hCG treatment decreased hippocampal (NS 44.5 ± 3.9% vs. hCG 28.5 ± 3.8%) and striatal (NS 23.5 ± 3.1% vs. hCG 16.7 ± 2.3%) tissue loss following HI. Actual area values (average ± SEM mm^2^) in left vs. right hippocampus are 1.8 ± 0.12 vs. 0.97 ± 0.10 mm^2^ for NS and 1.8 ± 0.14 vs. 1.3 ± 0.10 mm^2^ for hCG. Average area values in left vs. right striatum are 3.3 ± 0.19 vs. 2.4 ± 0.12 mm^2^ for NS and 3.0 ± 0.16 vs. 2.5 ± 0.12 mm^2^ for hCG. **(B)** Representative coronal digital micrographs taken from injured NS- and hCG-injected animals treated 15 h prior to HI. Note the ex-vacuo dilatation of the left, ischemic, cerebral ventricle, and relative atrophy of the left hippocampal formation in the NS group compared with the contralateral brain. **(C,D)** IP injection of hCG (1,500 IU/kg) 1 h **(C)** or immediately after **(D)** neonatal injury in P7 neonatal pups did not protect against the neurodegenerative effects of HI. Error bars show SE; *p < 0.05 and **p < 0.01. hCG, human chorionic gonadotropin; HI, hypoxia-ischemia; Hipp, hippocampus; IP, intraperitoneal; NS, normal saline; Str, striatum.

Term-equivalent C57BL/6 mouse pups were subjected to unilateral carotid ligation followed by 45 min of 8% hypoxia. As shown in Figures 1A,B, IP administration of 1,500 IU/kg of hCG 15 h prior to neonatal global cerebral hypoxia resulted in a 36% reduction in the amount of hippocampal tissue loss (% change in left vs. right brain; NS: 44.5 ± 3.9; hCG: 28.5 ± 3.8; *p* = 0.008) and a 29% reduction in striatal tissue loss (% change in left vs. right brain; NS: 23.5 ± 3.1; hCG: 16.7 ± 2.2; *p* = 0.049) 7 days after HI compared to saline-injected littermate controls. Next, 1,500 IU/kg of hCG or saline control solution was injected into P7 pups either 1 h prior to hypoxia or immediately after hypoxia exposure. As shown in Figures 1C,D, IP administration of hCG given at these two time periods did not result in protection against cerebral degeneration following HI in any of the cerebral regions examined. We did not observe any deaths associated with hCG or saline injection and there were no significant changes in pup weight between control animals (4.10 ± 0.1 g; *N* = 32) and hCG-treated animals (4.08 ± 0.1 g; *N* = 30).

### hCG Exposure Does Not Affecting Body Temperature

**Figure 2:**
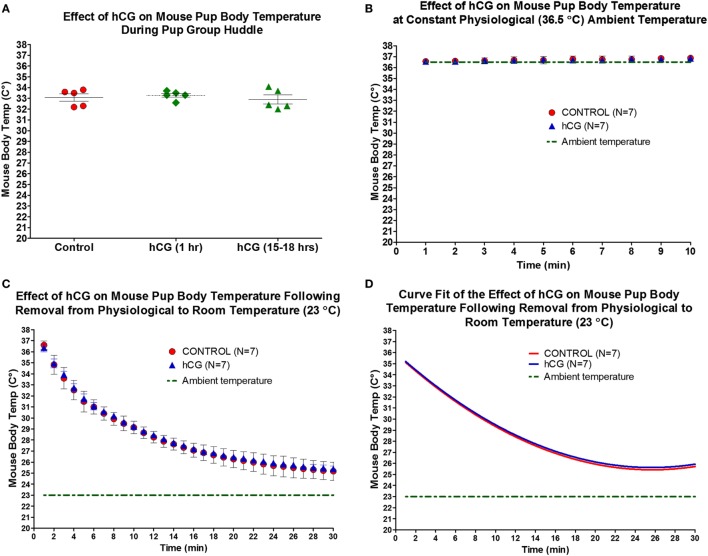
IP administration of hCG (1,500 IU/kg) does not alter neonatal pup body temperature. **(A,B)** Body temperature as assessed by rectal temperature in neonatal P7 mouse pups did not change 1 h or 15–18 h following hCG injection when examined during pup huddling or during a constant physiological ambient temperature of 36.5°C. **(C)** Removal of individual neonatal P7 pups from physiological (36.5°C) to room temperature (23°C) produced a gradual decline in body temperature. hCG administration did not alter the rate of temperature decline upon ambient temperature exposure when examined 15–18 h after drug injection. Statistical comparisons were performed by repeated measures ANOVA followed by Bonferroni *post hoc* test. **(D)** Second-degree polynomial curve fit calculated from data in **(C)**, demonstrating nearly identical temperature drops upon change in ambient temperature from 36.5 to 23°C in NS control and hCG-treated neonatal P7 pups. hCG, human chorionic gonadotropin; IP, intraperitoneal.

Given that hCG receptors are implicated in the regulation of the central hypothalamic-pituitary-gonadal axis in adult rodents (11), and understanding that neonatal hypothermia has been shown to reduce cerebral tissue loss following HI (45), we asked whether the neuroprotective actions of peripheral hCG administration are attributed to a decrease in body temperature. Intraperitoneal injection of 1,500 IU/kg of hCG 1-h or 15–18-h post-hormone injection did not alter neonatal P7 pup temperature when temperature was obtained in mouse huddle conditions compared with saline-treated littermates (Figure 2A; saline control: 33.08 ± 0.34°C; 1 h post-hCG: 33.28 ± 0.18°C; 15–18 h post-hCG: 32.90 ± 0.42°C; *N* = 5/condition). Similarly, pup body temperature in mice exposed to a constant physiological ambient temperature of 36.5°C did differ between mice exposed to saline and those exposed to 1,500 IU/kg of hCG 15–18 h prior to temperature recording (Figure 2B; saline control: 36.7 ± 0.1°C; 15–18 h post-hCG: 36.6 ± 0.1°C; *N* = 7/condition). Since neonatal mouse body temperature is highly dependent on ambient temperature (46, 47), we wanted to rule out the possibility of hCG altering the adaptation of the mouse neonate to rapid changes in ambient temperature. To examine this question, paired 15–18 h post-hCG- and saline-injected mouse pups were rapidly transitioned from a 30-min period of constant physiological ambient temperature of 36.5°C to room ambient temperature (23°C) while body temperature was continuously recorded for 30 min. Under these conditions, pup temperature gradually declined to a nearly identical degree and pattern between hCG and saline-treated groups (Figures 2C,D). We did not examine hCG’s effect on brain temperature prior and following HI and thus, a confounding effect of hCG on cerebral temperature in the injured neonate cannot be conclusively ruled out. Nevertheless, the presented data above suggest that hCG alone does not affect pup body temperature. Therefore, it is less likely that intrinsic differential effects of hCG on body temperature contribute significantly to the neuroprotective effect observed upon peripheral injection of hCG 15 h prior to HI.

Importantly and as shown in Figure 2C, the body temperature of P7 pups is tightly dependent on ambient temperature. Therefore, transient secondary hypothermia can occur when animals are removed from the hypoxia chamber and placed back with the dam. In order to minimize this potential confounder, hCG- and saline-treated animals were exposed to the hypoxia-chamber simultaneously and were placed back with the dam following hypoxia as a group while making every attempt to have an equal proportion of control and hCG-treated pups in each litter. Qualitatively, we did not observe differential treatment in the behavior of the mother toward either treatment condition with the dam resuming nesting and feeding of her pups almost immediately after being placed back in their cage.

### Serum hCG Levels 1 and 15–18 h Post 1,500 IU/kg hCG Injection are Similar to Those Found in the Amniotic Fluid of Term Human Neonates

**Figure 3:**
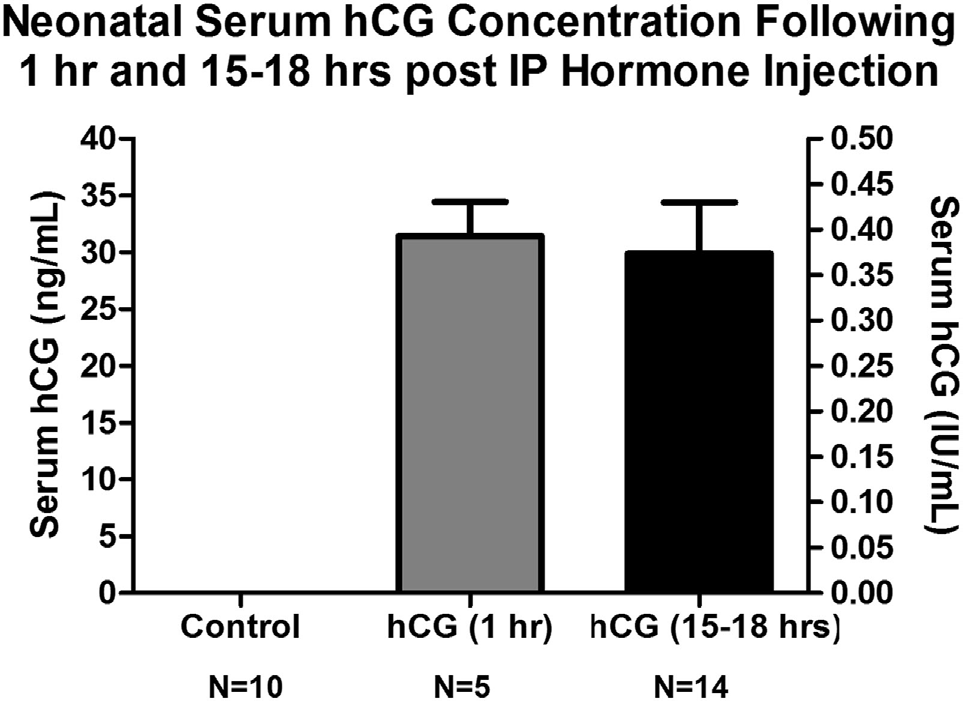
Serum hCG concentrations obtained from neonatal P7 mouse pups 1 and 15–18 h following IP 1,500 IU/kg hCG injection. Serum hCG levels (calculated in ng/mL) were obtained utilizing a commercially available hCG ELISA assay (ab100533, Abcam, Cambridge, MA, USA). Conversion to IU/mL was done using this assay and a known concentration of the hCG source employed in the animal and cell experiments (CG10, Sigma, St. Louis, MO, USA). No hCG IR was observed in control, NS-injected, mouse serum indicating no mouse protein cross-reactivity. The serum hCG concentration did not appear to differ significantly between 1 h (31.4 ± 3 ng/mL; 0.41 ± 0.04 IU/mL) and 15–18 h (29.8 ± 4 ng/mL; 0.39 ± 0.06 IU/mL) post-IP hormone injection. These serum concentrations are similar to those found in human term amniotic fluid (43, 48). hCG, human chorionic gonadotropin; IP, intraperitoneal, IR, immunoreactivity; NS, normal saline.

In order to have a better understanding of how the hCG dose used in this study relates to the serum levels commonly found in human cord blood and amniotic fluid, we extracted serum from the blood of P7 animals injected with 1,500 IU/kg of hCG 1 and 15–18 h after hormone injection and determined their serum hCG levels (in ng/mL and IU/mL) utilizing a commercially available serum hCG ELISA assay. As shown in Figure 3, the mean ± SEM serum hCG concentrations in the 1 and 15–18 h post-hCG groups were 31.4 ± 3 and 29.8 ± 4 ng/mL, respectively. These levels corresponded to 0.41 ± 0.04 IU/mL (1 h post-hCG) and 0.39 ± 0.06 IU/mL (15–18 h post-hCG) when the internal standard curve of the ELISA assay was compared with a dilution curve generated by the hCG used in this study’s animal and *in vitro* experiments. Although the concentration of hCG in cord blood and amniotic fluid varies according to gestational age, prior studies report cord blood and amniotic fluid hCG levels in term neonates to be around 0.2 and 0.4 IU/mL, respectively, with amniotic fluid levels averaging about 2 IU/mL during the third trimester of pregnancy (43, 48). Taken together, the above serum hCG levels found following a single-IP hCG dose of 1,500 IU/mL appear to be within the range observed in humans.

### Chronic *In Vitro* Exposure of Immature Cortical and Hippocampal Neurons to hCG Protects against NMDA-Dependent Excitotoxic Injury

**Figure 4:**
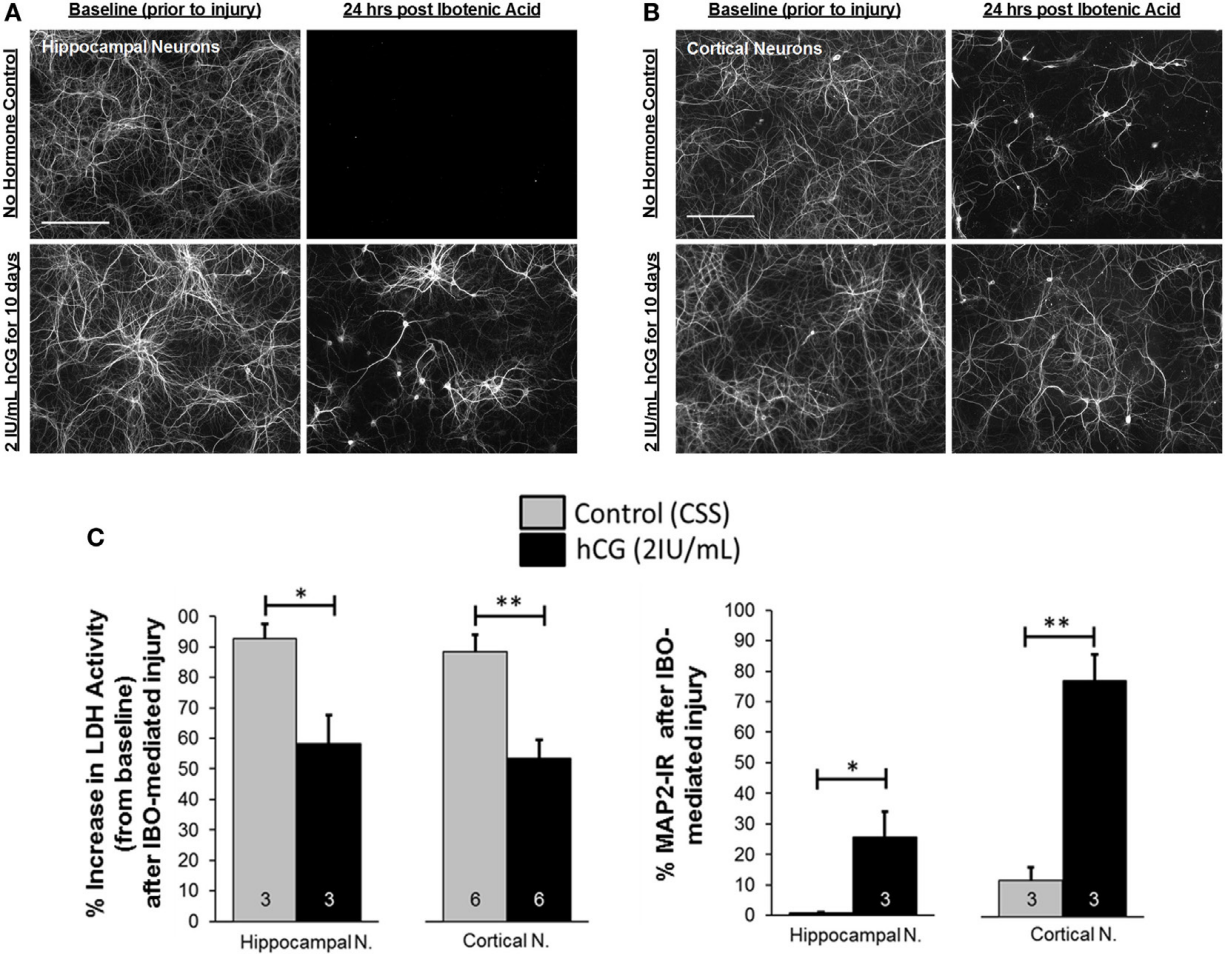
*In vitro* chronic, 10-day, hCG exposure to immature cortical and hippocampal neurons is not toxic and protects them from NMDA-dependent excitotoxic injury. **(A,B)** MAP2 fluorescence following immunocytochemical staining of dissociated cortical and hippocampal neurons exposed to control vs. 2 IU/mL of hCG for 9 days prior and 24 h following IBO (50 µM; IBO) exposure. Prolonged hCG did not appear to affect neuronal survival. Note also the relative preservation of neurites in hCG-exposed neurons after IBO exposure (most noticeable in cortical neurons) compared with control (CSS; controlled salt solution). **(C)** Quantitative bar graph analysis of the effects of hCG on neuronal survival as a measure of LDH activity in the neuronal media or MAP2 IR in control and hCG-treated neuronal cultures 24 h after injury with IBO. Cortical and hippocampal neurons exposed to hCG demonstrated a reduction in IBO-mediated increases in LDH activity and a relative preservation of neurite staining compared with non-hCG-treated cells. Scale bars = 165 µm. Error bars show SE; *p < 0.05 and **p < 0.01. hCG, human chorionic gonadotropin; IBO, ibotenic acid; IR, immunoreactivity; LDH, lactase dehydrogenase.

We then investigated whether chronic (10-day) exposure of immature neurons to 2 IU/mL hCG, a concentration of hormone found in human amniotic fluid (42–44), protects against NMDA-mediated excitotoxic neurodegeneration. Chronic hCG exposure resulted in a significant decrease in neuronal NMDA-mediated excitotoxicity; this is demonstrated by the relative increase in the percent of MAP2 immunoreactivity (IR) in hippocampal (0.79 ± 0.3 vs. 25.5 ± 8.4%; *p* = 0.021) and cortical (11.74 ± 4.4 vs. 77.1 ± 8.8%; *p* = 0.001) neuronal cultures 24 h following excitotoxic insult with IBO (Figures 4A–C) as compared with non-hCG exposed neurons. Similarly, hippocampal and cortical cultures chronically exposed to hCG demonstrated a significant decrease in the percent elevation of LDH activity in the culture media 24 h following IBO exposure (92.6 ± 4.8 vs. 58.3 ± 9.4%; *p* = 0.015; and 88.4 ± 5.4 vs. 53.3 ± 6.2%; *p* = 0.001, respectively; Figure 4C). The combination of these findings (increased neurite preservation and decreased LDH activity) suggests that prolonged exposure (beginning before injury) of hCG (2 IU/mL) directly to immature dissociated brain neurons is capable of decreasing NMDA-mediated excitotoxic neurodegeneration.

### Acute *In Vitro* hCG Exposure Protects Neurons against NMDA-Mediated Excitotoxic Neurodegeneration

**Figure 5:**
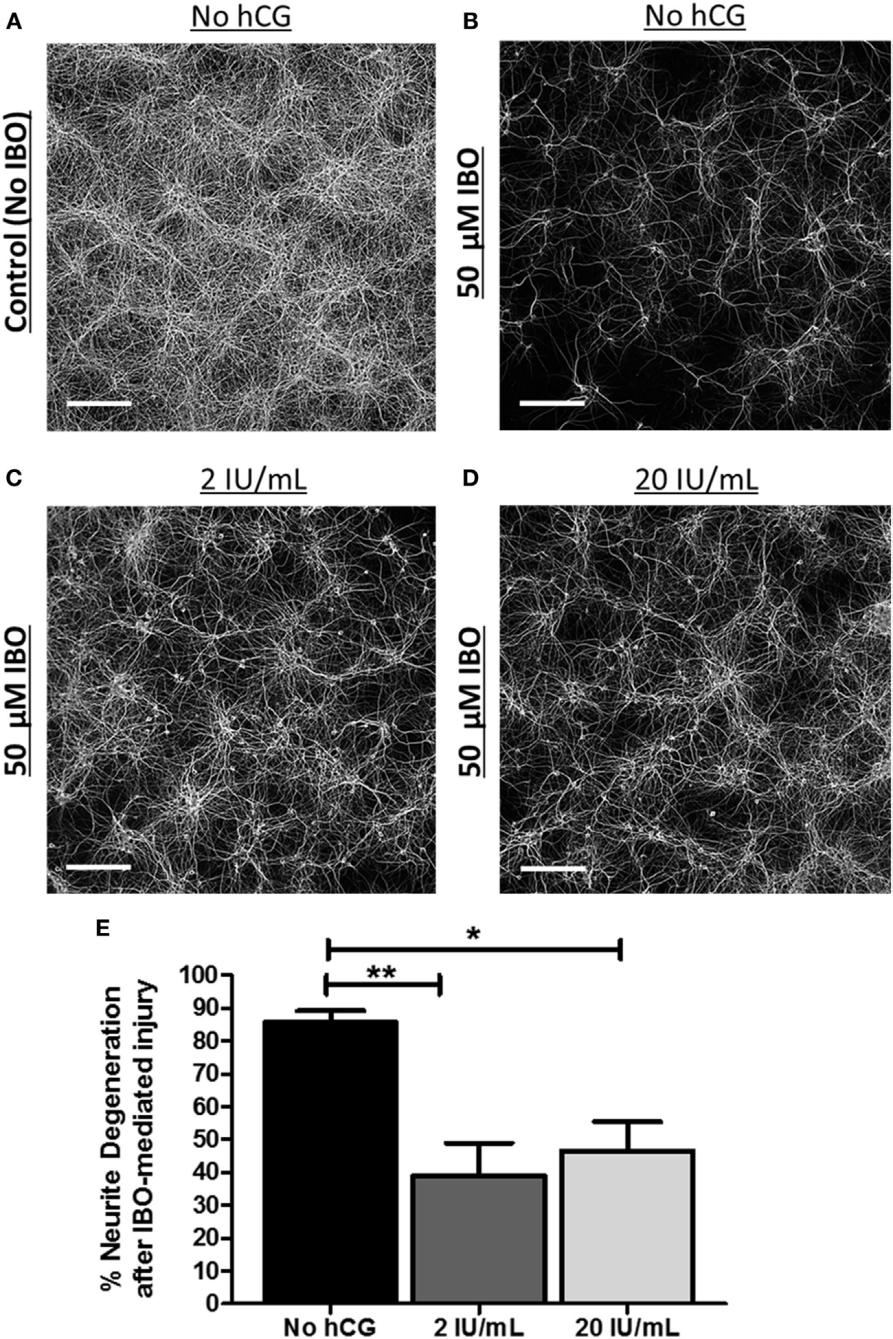
Acute neuronal hCG exposure *in vitro* protects neurons against NMDA-mediated excitotoxic neurodegeneration. **(A)** Representative low-power digital micrograph of MAP2 IR in DIV12 cultured, non-injured/non-hCG-treated, cortical neurons. **(B–D)** Representative digital micrographs of DIV12 cultured MAP2-stained neuronal fibers 24 h following exposure to the NMDA-receptor agonist, IBO in the presence or absence of 2 or 20 IU/mL of hCG when hCG is administered during and following IBO exposure. **(E)** Quantitative summary of the neuroprotective effect of acute hCG administration 24 h post-IBO administration. Percent neurite degeneration was obtained as a function of the percent change in MAP2-IR from non-IBO control neurons. Scale bar = 220 µm. *N* = 4/condition. Error bars show SE; *p < 0.05 and **p < 0.01. hCG, human chorionic gonadotropin; IBO, ibotenic acid; IR, immunoreactivity.

We then investigated whether acute exposure of immature neurons to low-dose hCG (2 IU/mL) or to high-dose hCG (20 IU/mL) protects against NMDA-mediated excitotoxic neurodegeneration. hCG administered at the time and 24 h following IBO exposure significantly reduced IBO-mediated cortical neurite neurodegeneration by 55% (*p* < 0.01) at 2 IU/mL hCG and by 46% (*p* < 0.05) at 20 IU/mL hCG when compared with their respective non-hCG-treated injured neuronal controls (Figures 5A–E). The neuroprotection seen at 2 IU/mL hCG concentration vs. that seen at 20 IU/mL hCG concentrations was not significantly different from each other (Figure 5E). These data indicate that hCG, when administered directly to neurons at the time of injury is able to reduce NMDA-mediated neuronal excitotoxicity.

### Early (DIV3) High-Dose hCG Exposure *In Vitro* Increases Basal Neuronal Survival

**Figure 6:**
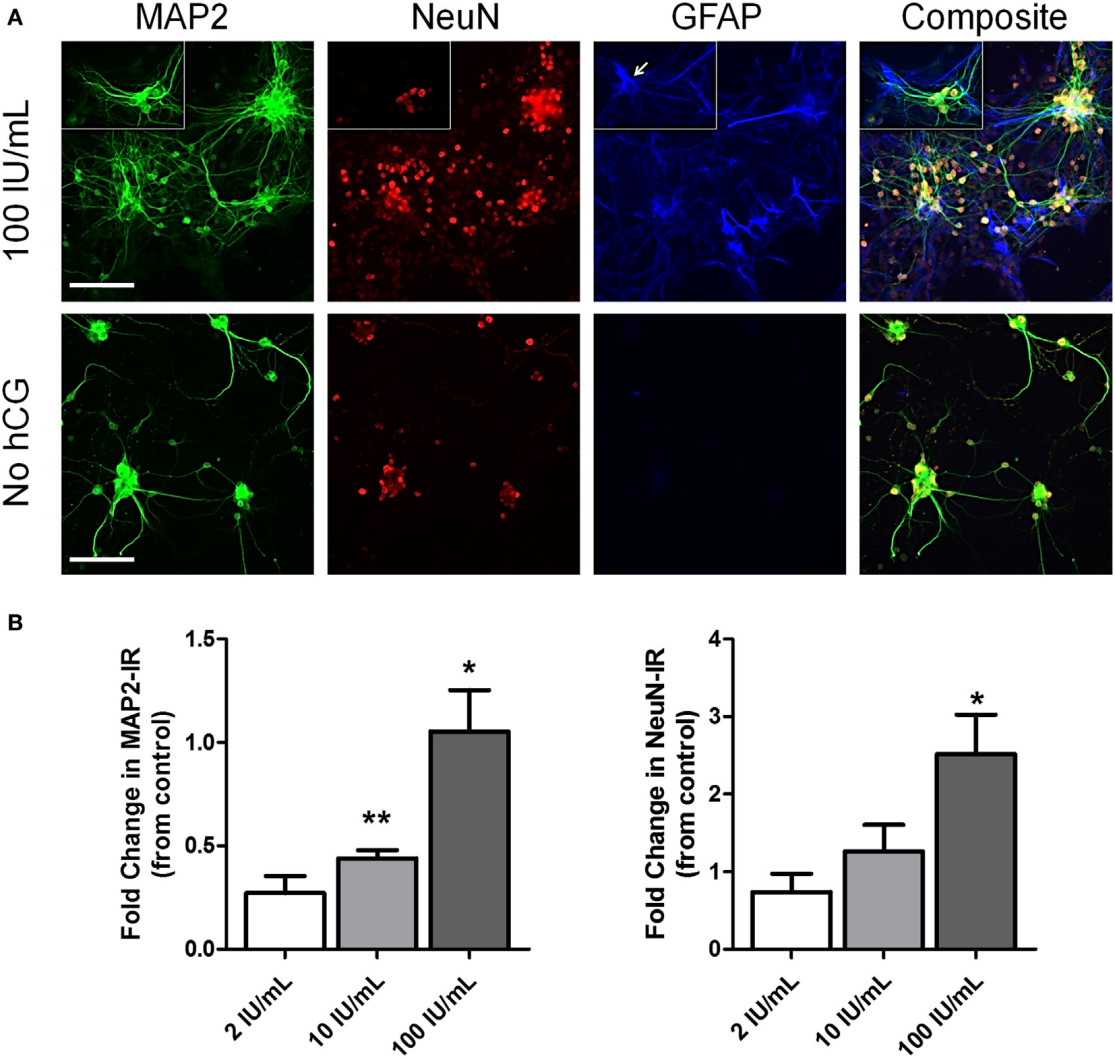
Early (DIV3) high-dose hCG (100 IU/mL) exposure *in vitro* increases neuronal cell count and neurite sprouting. **(A)** Representative low-power micrographs of DIV9 cortical neurons exposed to high-dose (10 and 100 IU/mL) hCG for 6 days and triple-stained with the neurite marker, MAP2, the neuron-specific marker, NeuN, and the astrocyte-specific marker, GFAP. Prolonged high concentrations of hCG resulted in large grouping of neurons with more complex neurite processes surrounded by GFAP + astrocytes. In contrast, GFAP + astrocytes are nearly absent in non-hCG-treated cultures at DIV9. Small rectangular inserts demonstrate a morphologically appearing GFAP + astrocyte (arrow) nearby a small group of NeuN + neurons. **(B)** A significant increase in MAP2 IR and visible increases in neuronal sprouting were observed with greater than 2 IU/mL concentrations of hCG. High-dose hCG (100 IU/mL) appeared to qualitatively and quantitatively increased NeuN + IR. Scale bar = 80 µm. *N* = 3/condition. Error bars show SE; *p < 0.05 and **p < 0.01. hCG, human chorionic gonadotropin; IR, immunoreactivity.

We also studied the potential neurotrophic effects of hCG by examining basal neuronal survival of immature cortical neurons exposed to high concentrations of hCG starting at DIV3. Six-day hCG treatment at 100 IU/mL concentration resulted in a visible (Figure 6A) and quantitative (Figure 6B) 1.05 ± 0.2-fold (*p* = 0.034) increase in neurite sprouting as seen in MAP2 immunofluorescence staining and a quantitative 2.5 ± 0.5-fold (*p* = 0.038) increase in neuronal cell count as seen in NeuN immunofluorescence staining.

Neuronal MAP2 + cell processes in high-dose hCG-treated neurons appeared longer and more complex suggesting the possibility of an hCG-mediated increased in neurite sprouting (Figure 6A). Interestingly, we also observed daily progressive grouping of neurons surrounded by morphologically appearing astrocytes only in the cultures exposed to high 100 IU/mL hCG. Triple staining of these cultures with MAP2, NeuN, and GFAP confirmed the increased presence of GFAP + astrocytes 6 days following high-dose hCG exposure. In contrast, GFAP + staining was almost completely absent in non-hCG-treated neurons at this time in culture (Figure 6A). Similarly, in a separate experiment utilizing a different source of hCG, we observed a significant 1.3 ± 0.36-fold (*p* = 0.002; *N* = 3; graph not shown) increase in MAP2-IR following continuous 9-day hCG administration at a concentration of 500 ng/mL. Taken together, these data support the conclusion that prolonged, direct exposure of neurons to high hCG concentrations under basal conditions promotes neuronal survival and potentially increases neuronal sprouting.

## Discussion

The *in vivo* study results suggest that hCG decreases HI brain injury when the neonate is exposed to hCG before the hypoxic-ischemic event occurs. When hCG was peripherally administered many hours (≥15 h) before HI induction, its effect against neonatal hippocampal and striatal injury was clearly evident. Since the human fetus is chronically exposed to placentally derived hCG throughout its intrauterine life, hCG may potentially play a physiological role in protecting the developing brain from hypoxic-ischemic events that occur *in utero* or around the time of birth.

In order to examine the *in vivo* neuroprotective actions of hCG, we chose the Rice–Vannucci model of neonatal HI for this study because this model has been widely used by researchers to examine perinatal hypoxic-ischemic brain injury (49–51). This model is unique in that its combination of unilateral carotid occlusion and the systemic hypoxia produces the type of hypoxic-ischemic brain injury in limbic (hippocampus) and basal ganglia (striatum) regions that can be observed in moderately to severely affected human neonates. However, this injury model is only a functional model of neonatal injury and a limitation of this paradigm is that it does not truly simulate the exact environmental and physiological parameters that are seen in newborn HI. Furthermore, the degree of injury used in our model does not induce robust tissue loss in the cortex (35). This is also a limitation of the employed injury paradigm because hypoxic-ischemic events in the human fetus/newborn do often include the cerebral cortex (52). Perhaps the relatively small amount (approximately 5%) (35) of cortical tissue loss after HI induction observed explains the apparent lack of protection by hCG against the cortical injury seen in this study.

Because hypothermia is known to reduce cerebral injury after HI (45, 53) and central hCG receptors are known to affect hypothalamic function (11) (a brain region importantly involved in core body temperature regulation), we examined whether hCG’s neuroprotective actions are attributed to a decrease in body temperature. We confirmed the previous observation (54) demonstrating that the body temperature of P7 neonates is highly dependent on ambient temperature with rapid decreases in pup temperature observed upon transfer of the neonates from 37 to 23°C; this effect has the potential to cause unwanted hypothermia and alter the degree of cerebral injury. Importantly, we found that IP administration of hCG does not affect the body temperature of healthy P7 neonates. Nevertheless, we did not examine body temperature prior and after hCG injection in the injured newborn mouse. Neonatal HI has been shown to decrease body temperature in rodents (55). Therefore, we cannot rule out the possibility that in the setting of HI, hCG potentiates a further drop in body temperature which, in turn, could contribute to the observed decrease in cerebral tissue loss. The potential contribution of hCG to mouse temperature during and after brain injury should be explored in future studies in order to better understand the neuroprotective effect of hCG found in this report.

Regarding the neonatal age examined, though induction of HI sooner after birth (such as on postnatal 5) may have altered the degree of cortical injury, we chose to follow a more common protocol for this model which involves induction of HI at P7. Of note, this age in a mouse most closely approximates the developmental stage of late preterm human infants. Future studies can examine brain injury at postnatal days before and after this time point to assess whether hCG has differential neuroprotective properties depending on the stage of brain development. In this study, we also evaluated the brains of mouse pups at 7 day post-injury. Since brain injury may potentially still be evolving at this time, future studies should also examine the long-term effects of hCG-mediated protection to affirm that the neuroprotection is permanent. The reason that we also tested hCG after HI induction was to determine whether there may be a potential benefit for hCG administration to an infant postnatally if an HI event occurs shortly after birth. However, our results do not suggest that this is the case since we did not see any reduction in brain injury when hCG was administered shortly before or immediately after the induction of HI. Therefore, our findings suggest that the neurological effects of hCG may only be protective and not reparative. Perhaps once neurodegenerative cellular processes have already been triggered, hCG exposure is ineffective. It is also possible that hCG may offer beneficial effects against HI-mediated cerebral injury that were not detected by the hCG dose examined by our outcome measure (i.e., percentage of region-specific tissue loss). An alternative explanation of the apparent lack of effect when administered right before or after injury may be related to the mode of hCG administration. Available human and animal data indicate that peak blood levels of hCG are not achieved until at least 3 h following intramuscular administration (which is known to be a faster route of delivery to that of the IP route) (36, 56–59). Therefore, pharmacologically relevant hCG actions on the brain may not begin until hours after IP administration. hCG treatment immediately post HI or 1 h prior to neonatal injury may thus not accurately reflect the full protective or potential restorative actions of this hormone in the injured brain.

The difficulty over the years in defining a possible physiologic role of hCG in human fetal development has been partially attributed to a lack of suitable animal models. In humans, both (placentally derived) hCG and (pituitary-derived) luteinizing hormone (LH) bind to the same lutropin receptor (LHR) (1, 44). Despite some biological and functional differences between these hormones, hCG is highly analogous to LH and is often regarded as a super powerful form of it. Lutropin receptors have been identified in almost all species ranging from rodents to human (60, 61). However, the presence of two different LHR ligands (LH and hCG) is primarily a human phenomenon; most mammals (except for humans, primates and horses) produce only LH (44). Given that rodents do not naturally produce any type of chorionic gonadotropin, rodent models are not relevant models in distinguishing physiologic vs. pharmacologic levels of hCG; unlike human fetuses/neonates, no dosage of hCG is physiological to a neonatal rodent. That said, we find that the 1,500 IU/kg dose of hCG administered intraperitoneally in this study is a close approximation to the hCG levels seen in human amniotic fluid at term (43). We justify the use of the rodent model in this study as a way to determine a possible neuroprotective role for hCG in fetal development and not to establish the dosage that would be required to achieve the effect.

In the second part of the study, we directly explored the actions of hCG on neuronal degeneration following *in vitro* neuronal injury. The pathologic process of excitotoxicity occurs when glutamate receptors, such as NMDA receptors, are overactivated; excessive NMDA receptor stimulation is known to result in neuronal damage or death *via* rapid increases in intracellular calcium (17, 62, 63). NMDA-mediated excitotoxicity plays a pathophysiological role in a wide range of other brain-injury paradigms such as following focal or global cerebral ischemia (17, 63, 64). In particular, glutamate-dependent excitotoxicity is a contributor to the pro-degenerative mechanisms involved in neonatal HI (63, 64). For example, HI-mediated brain injury in term-equivalent neonatal rats can be significantly reduced by pre-treatment with the NMDA antagonist, MK801 (65, 66). Conversely, overactivation of neuronal NMDA receptors *in vitro* by NMDA, or *in vitro* and *in vivo* by the NMDA-agonist, IBO, results in dose-dependent cerebral and neuronal degeneration (40, 67).

Given the above, we utilized the well-characterized model of *in vitro* NMDA-mediated neuronal excitotoxicity to examine whether hCG inhibits excitotoxic neuronal degeneration of immature cortical and hippocampal neurons. In this model, inhibition of excitotoxic neuronal degeneration is measured by a decrease in LDH activity accompanied by a decrease in percentage of injury-triggered neurite loss. Importantly, our study shows that hCG protected neurons against glutamate-mediated excitotoxicity after prolonged hCG exposure as well as following acute drug exposure at the time of injury. Furthermore, our results suggest that hCG likely blocks NMDA-mediated damage to cell bodies as well as to neuronal processes, since degenerating neurites do not release LDH (68). Therefore, acute and prolonged *in vitro* hCG exposure protects neuronal cell bodies and their processes from the degenerative effects of NMDA-receptor overactivation. It is important to note, however, that cerebral injury mediated *via* glutamate receptor activation is only a partial contributor in the pathogenesis of cerebral HI and other cellular mechanisms may contribute to the neuroprotective actions of hCG as discussed below.

Remarkably, the amount of basal neuron and neurite density increased when cortical cultures were exposed to hCG concentrations higher than 2 IU/mL. This result is consistent with previous observations which indicate a dose-dependent increase in the number of neurite-bearing dissociated rat brain neurons following 3-day hCG exposure (6). Importantly, hCG contains domains encountered in the protein family of neurotrophic glycoproteins (69). Thus, the observed hCG-dependent increases in MAP2-stained fiber density may be indicative of hCG’s potential neurotrophic actions on immature neurons. It will be important in future studies to determine (a) which signaling pathways are activated in the brain by hCG, (b) which receptors are required for neuroprotective effects, and (c) whether neuroprotective effects are due to cell autonomous vs. non-cell autonomous mechanisms.

All in all, the results of this study enhance our understanding of the potential physiologic role of hCG in neuroprotection during brain development. The findings described in this report suggest that hCG is capable of decreasing the injury of immature neurons *in vivo* (prophylactically) and *in vitro*. In addition, our findings show that peripheral hCG exposure prior to HI is required to decrease neuronal injury. This raises the possibility that preterm infants, at higher than normal risk for HI-simulated events postnatally, may benefit from prophylactic administration of hCG. After all, preterm infants are prematurely deprived of placental hCG exposure due to their early separation from placentally derived hormones. In other words, the developing brains of preterm infants would naturally have had longer exposure to hCG had they remained *in utero* until term. Future preclinical studies should be performed to further evaluate whether hCG supplementation to preterm infants at physiologically appropriate levels (to compensate for hCG deprivation) is beneficial. Of important note, administration of hCG at 1,500 IU/kg results in serum concentrations that have the potential to alter the levels of other hormones such as testosterone. Thus, further preclinical and clinical studies will be needed to determine whether there is an hCG dose that is effective at providing neuroprotection to injured neonates while producing minimal potentially adverse systemic effects if it is to be considered for clinical use.

Our results also suggest that one of hCG’s mechanisms of neuroprotection may be related to its inhibition of NMDA-dependent excitotoxic neurodegeneration. Yet, it is important to emphasize that hCG has multiple cellular targets in peripheral tissues and brain (11, 44). Therefore, the neuroprotective actions of hCG may be multifactorial and thus, it is unlikely that hCG’s ability to reduce glutamate-mediated excitotoxic injury is its only mechanism of action. For example, hCG may further ameliorate the effects of cerebral injury in preterm and term newborns by dampening systemic and/or central inflammation. Emerging research in human reproduction demonstrates that one of hCG’s important roles in pregnancy is to modulate the maternal immune response and prevent the rejection and demise of the semi-allogenic fetus (70). hCG modulates the phagocytic action of macrophages, dampens the pro-inflammatory activity of T-lymphocytes, inhibits B-cell antibody production and suppresses the synthesis of pro-inflammatory cytokines such as TNF-α and interferon-gamma (71–77). hCG also increases the synthesis of anti-inflammatory cytokines such as IL-10 (76), as well as activates the production of pro-survival immunomodulatory cytokines such as leukemia inhibitory factor (78–82). Interestingly, all the above hCG-regulated proteins play a very important role in the pathogenesis of cerebral white matter degeneration of the newborn, a condition that significantly contributes to the development of CP in term and preterm neonates (83). Therefore, future studies should be performed to better characterize the systemic and central-nervous-system-mediated mechanisms that may contribute to the potentially beneficial effects of this hormone against the effects of early cerebral injury.

## Ethics Statement

Animal care and use in our laboratory were in strict accordance with the National Institute of Health guidelines on the use of laboratory animals. All procedures were also approved by the Animal Studies Committee at Washington University.

## Author Contributions

TZM and RG drafted the manuscript and made substantial contributions to the conception, experimental design, acquisition, analysis and interpretation of the scientific data. RLW, MBG and DMH contributed to the intellectual content of this work, and they were involved in the acquisition, analysis and/or interpretation of the data. All authors have given approval to the final version of the manuscript and all authors agreed to be accountable for all aspects of the work. We thank Tyler Frank and Alexia Robinson for the technical contributions to this publication.

## Conflict of Interest Statement

No payment was received from any third party for any aspect of this study. TZM (of Zietchick Research Institute, a drug discovery start-up) and RG (of Washington University) have jointly filed a patent application for the use of hCG for the prevention of cerebral palsy and its comorbidities which is currently pending. All other authors declare that the research was conducted in the absence of any commercial or financial relationships that could be construed as a potential conflict of interest.
